# Ghost cell odontogenic carcinoma: A rare case report and review of literature

**DOI:** 10.4317/jced.51809

**Published:** 2014-12-01

**Authors:** Míriam Martos-Fernández, Margarita Alberola-Ferranti, Juan Antonio Hueto-Madrid, Coro Bescós-Atín

**Affiliations:** 1 MD. Resident, Oral and Maxillofacial Surgery Department, Vall d’Hebrón Hospital. Barcelona, Spain; 2 PhD, MD. Department of Pathology, Vall d’Hebrón Hospital. Barcelona, Spain; 3 MD, DDS. Assistant Surgeon, Oral and Maxillofacial Surgery Department, Vall d’Hebrón Hospital, Barcelona, Spain. Researcher of the VHIR group; 4 PhD MD, DDS. Head of Oral and Maxillofacial Surgery Department, Vall d’Hebrón Hospital. Barcelona, Spain. Researcher of the VHIR group

## Abstract

Objectives: Ghost cell odontogenic carcinoma is a rare condition characterized by ameloblastic-like islands of epithelial cells with aberrant keratinitation in the form of Ghost cell with varying amounts of dysplastic dentina. Material and Methods: We report a case of a 70 year-old woman with a rapid onset of painful swelling right maxillary tumor. Magnetic resonance showed a huge tumor dependent on the right half of the right hard palate with invasion of the pterygoid process and focally to the second branch of the trigeminal. Radiological stage was T4N0. The patient underwent a right subtotal maxillectomy with clear margins. Adjuvant radiotherapy was given. The patient was free of residual or recurrent disease 12 months after surgery. Results: The tumor was 3,9cm in diameter. It was spongy and whitish gray. Microscopically the tumor was arranged in nets and trabeculae, occasionally forming palisade. Tumoral cells had clear cytoplasm with vesicular nuclei. There was atipia and mitosi with vascular and perineural invasion. The excised tumor was diagnosed as a GCOC. Conclusions: Ghost cell carcinoma is a rare odontogenic carcinoma. Its course is unpredictable, ranging from locally invasive tumors of slow growth to highly aggressive and infiltrative ones. Wide surgical excision with clean margins is the treatment of choice although its combination with postoperative radiation therapy, with or without chemotherapy, remains controversial.

** Key words:**Ameloblastic carcinoma, calcifying odontogenic cyst, Ghost cell carcinoma, keratinizing epithelial odontogenic cyst, maxillary tumor, odontogenic carcinoma.

## Introduction

Ghost cell odontogenic carcinoma [GCOC] is a rare malignant odontogenic epithelial tumor with features of calcifying odontogenic cysts that can arise as a de novo tumor or from a previously existing calcifying cystic odontogenic tumor [CCOT] or dentinogenic ghost cell tumor [DGCT] after multiple recurrences (1). Its occurrence constituting about 0,37% to 2.1% of all odontogenic tumors (2). The GCOC was first described by Gorlin *et al.* in 1962 as a distinct pathological entity (3). Since then there have been approximately 30 cases reported in the literature to date. In 2005 The World Health Organization [WHO] classified them under the category of odontogenic tumours (4). The most characteristic histological feature is the presence of ghost cells, epithelial cells that have lost their nuclei leaving only a faint outline of the original nuclei. The presence of ghost cells is not a specific feature, they could be seen in others tumors such as pilomatricoma, craniopharyngioma, odontoma and ameloblastic fibro-odontoma (2).

This article reports a new case of GCOC in the right maxilla in a 70 year-old woman and describe its clinicopathological features, radiological images and treatment performed.

## Material and Methods

A 70 years old woman was referred by her dentist to our Oral and Maxillofacial Surgery Department at Vall d’Hebron Hospital in August 2013. She reported pain and rapid expansion of a mass located on the alveolar buccal surface of the right maxilla during the previous three weeks. She denied any clinical symptoms or lesions prior to this episode. No neurological deficit was referred to. The patient explained that initially was treated with Amoxiciline-Clavulanic acid 875mg/8h during one week due to the suspicious of periapical abscess but no im-provement was shown.

**Figure 1:**
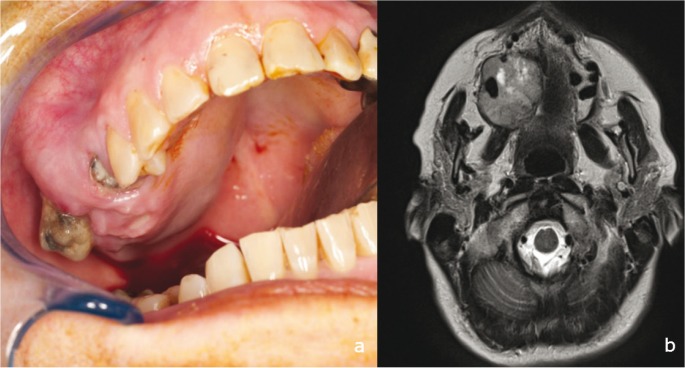
Clinical and MRI examination. a) Intraoral photograph showed an expansive non-fluctuant mass, ovoid in shape, on the right maxilla with palatine protrusion including 2nd premolar and 1st molar. Overlying mucosa was intact. b) MRI T1 before operation. Axial plane shows the tumor’s extension through the right side of the palate involving 2nd premolar and 1st molar without reaching the midline. The mass showed variable densities and bony erosion of the maxilla.

The patient was in good health. Extraoral examination did not show any pathological signs. The overlying skin was smooth and normal. No ulceration was observed. Enlarged cervical lymph nodes were not found on physical examination. Intraoral examination showed an expansive non-fluctuant mass of 5cm in the right maxilla with palatine protrusion involving the second premolar and first molar which presented mobility (Fig. 1).

- Complementary Tests:

A complete blood test was performed but no alterations were found. Chest x-ray showed no evidence of distant metastasis. Intraoral incisional biopsy was done under local anesthetic and the tissue was submitted to the Department of Oral Pathology. The first histopathological examination showed an infiltration by morphologically suggesting carcioma ameloblastic lesion.

The radiographic study also included an ortopantomograph and magnetic resonance imaging [MRI]. Panoramic radiograph demonstrated a large, poorly demarcated mixed radiopaque-radiolucent lesion with destruction of the buccal and palatine cortical plates associated with 15 and 16 root resorption without crossing the midline. MRI showed a huge expansive tumor committed to the right half of the palate and alveolar ridge side, but without reaching the midline. This lesion located at the level of molar area of the alveolar ridge was infiltrating the pterygoid process and invading the right nostril and the maxillary sinus. There was a tooth [first molar] included in the lump and also partially including the second premolar. It reached a maximum diameter of 40 x 32 x 34 mm. There seemed to be focally produced infiltration of the second trigeminal branch. No other changes in the elements of the oral cavity or significantly enlarged lymph nodes were observed. Radiological stage was informed as T4N0 (Fig. 1).

**Figure 2:**
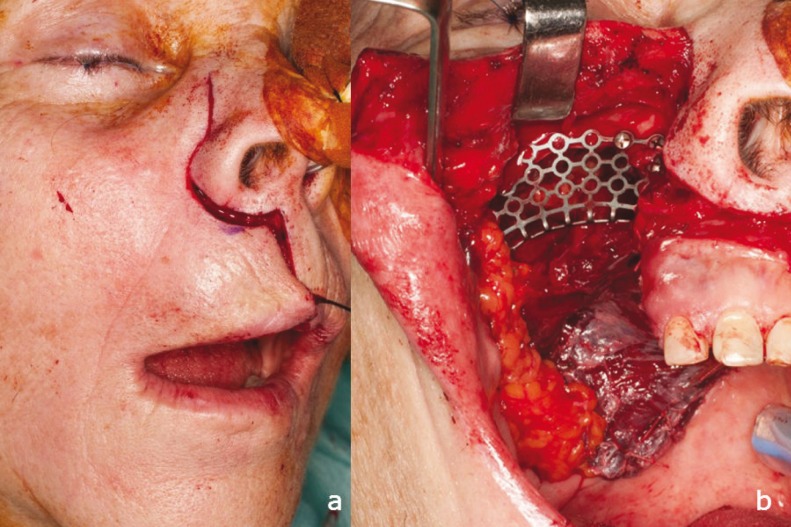
Tumor resection. a) Weber-Ferguson approach without infraorbital extension was performed. b) Right subtotal maxillectomy was conducted to achieve total tumor resection with clean margins. The defect was reconstructed with a Temporal rotation flap above an osteosynthesis plate in the right maxillary region to achieve greater malar projection.

- Treatment:

Based on these clinical findings surgical resection was decided under the diagnosis of ameloblastic carcinoma. The tumor was resected under general anesthesia with nasotracheal intubation by Weber-Ferguson approach without infraorbital incision extension (Fig. 2). An extended maxillectomy was performed from the maxillary tuberosity to the superior lateral incisor and the tumor was excised with clear margins. The defect left by the tumor was reconstructed with a temporal rotation flap over an osteosynthesis plate collocated in right maxillary region (Fig. 2). Bone cement was placed in the temporal fossa due to enhance the aesthetic results. Adjuvant radiotherapy treatment after surgery was performed during November 2013 [total cumulative dose: 52 Gy], and the patient was followed up for 12 months with no evidence of recurrence or distant metastasis.

**Figure 3:**
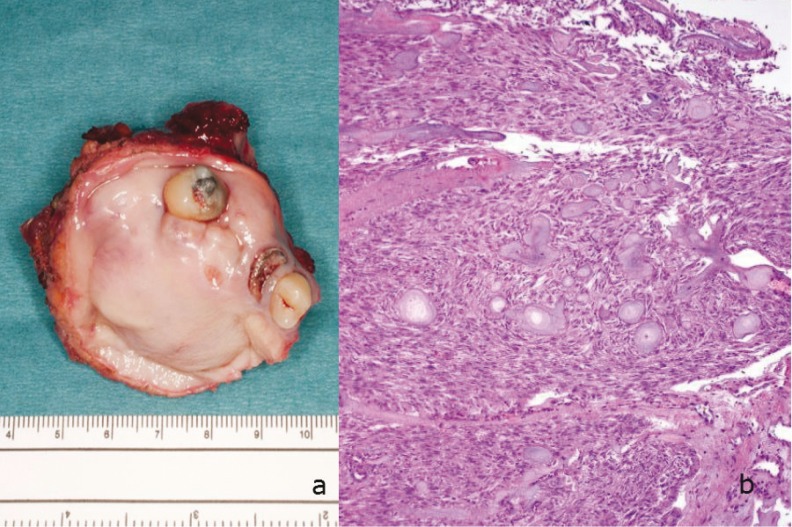
Pathological study. a) The total measurements of the sample sent for histopathological evaluation were 5.8 x 5.4 x 4cm and included palate and maxillary sinus floor. b) Photomicrograph of ameloblastic like islands and Ghost cells with odontogenic epithelium (H&Ex100).

Entire specimen was sent for histopathological evaluation. The sample measured 5.8 x 5.4 x 4cm in total and included palate and maxillary sinus floor (Fig. 3). The final outcome of the pathological study informed of a tumor lesion that consisted of a proliferation of cells with clear cytoplasm and vesicular nuclei, of ameloblastic appearance, which are arranged in large and irregular nests, with some peripheral lurch. Other smaller nests, hyperchromatic cells and atypia and mitosis were observed. Anucleated eosinophilic aggregates [Ghost cells] were trapped in the epithelium with concentric laminated structures and calcification (Fig. 3). The neoplastic cellularity of bone infiltrates palate and maxillary sinus floor. Neither the squamous surface of the palate nor the alveolar gingival epithelium was affected. Presence of perineural and vascular permeability was observed. In our case the diagnosis of GCOC was possible only after the resected specimen was carefully examined histologically.

## Discussion

GCOC is a rare malignant odontogenic tumor that although it can appear as “de novo” the most probable mechanism of development is a malignant transformation after multiple recurrences of a preexisting CCOT or other odontogenic tumor (5-7). Occasionally, some cases of Ameloblastoma associated with GCOC have been reported (8). According to the 2005 World Health Organization guidelines (4), GCOC is usually diagnosed on the basis of atypical histological features, groups of ghost cells, necrosis, prominent mitoses, infiltrative growth pattern, aggressive behavior, and high expression of Ki-67 and p53 (5). Several investigations show a high ki-67 and p53 expression in GCOC and a low one in CCOT (9-14). Nevertheless, further research is required to determine if these biomarkers of cell proliferation activity can be used to determine more probabilities of malignant transformation or high recurrence rate. The main differential diagnosis is ameloblastic carcinoma and the identification of Ghost Cell is the clue to diagnosis of Ghost cell odontogenic carcinoma.

Numerous names have been used to describe GCOC due to its diverse histopathologic characteristics. This tumor has been described as malignant CCOT, odontogenic ghost cell carcinoma, carcinoma arising in a CCOT, aggressive epithelial ghost cell odontogenic tumor, dentinogenic ghost cell ameloblastoma and malignant calcifying ghost cell odontogenic tumor. The odontogenic origin is widely accepted. The cells responsible for the CCOT are dental lamina remains within either the soft tissue or bone. Therefore, CCOT are cysts of primordial origin and are not associated with the crown of an impacted tooth (15). It most often occurs as an intraosseous lesion whereas peripheral extraosseous localization in the soft tissue is rare.

A review of the approximately 30 cases reported in the literature to date show that the average age is 40 years [13-72 years] with a male predominance, particularly in Asians (16,17). GCOC are more common in maxilla. It could cross the midline in the mandible but it is unusual in maxilla. The most common clinical presentation is a painful swelling with local paraesthesia associated with root resorption and/or tooth displacements. It may cause expansion of the mandible or maxilla. Its radiographic appearance is usually a mixed radiolucent and radiopaque pattern with different degrees of bone destruction and poorly defined [90%] rather than well-defined borders [11%] (18). Generally, its course is unpredictable, at times indolent and at others potentially fatal. Distant metastasis is extremely rare although to date 2 cases with pulmonary metastasis have been reported (16,19). The clinical features of previously reported GCOC and the present case are summarized in  Table 1.

**Table 1:**
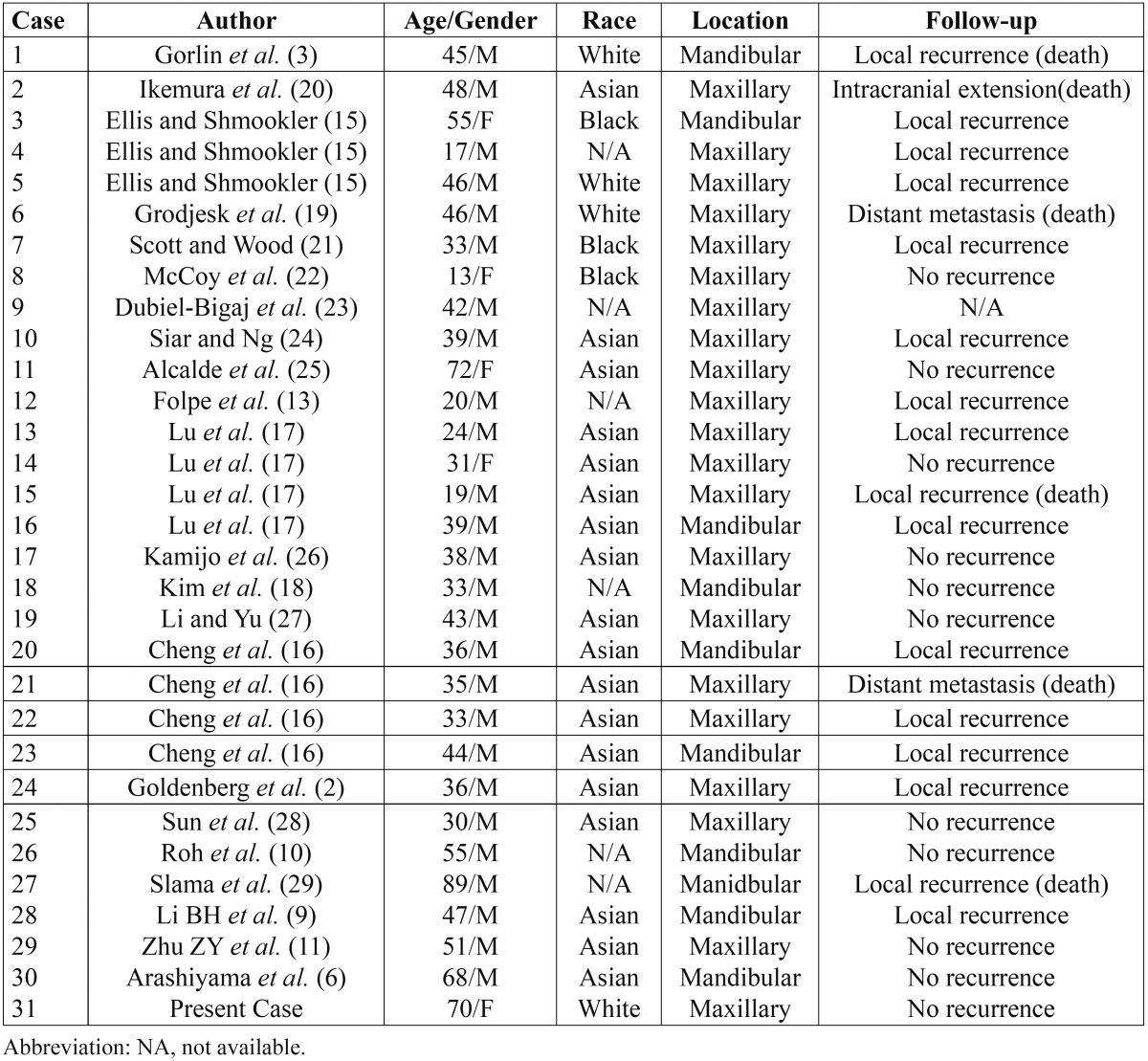
Clinical features of reported cases of odontogenic ghost cell carcinoma.

The recommended treatment for GCOC is wide surgical excision. Postoperative adjuvant irradiation, with or without chemotherapy, is controversial and any standard treatment has been evaluated. The overall five-year survival rate has been reported to be approximately 73% although long-term follow-up is highly recommended after therapy due to its unpredictable course (1).

## Conclusions

Ghost cell carcinoma is an uncommon odontogenic carcinoma. All reported cases demonstrated malignant histological features such as cellular pleomorphism, mitosis and necrosis with anucleated eosinophilic aggregates [ghost cells] in association with odontogenic epithelium. The biological behavior of GCOC is unpredictable, some cases are characterized by relatively indolent growth and others by aggressive behavior. A multidisciplinary team including a pathologist with expertise in evaluating odontogenic neoplasms is essential to determine proper treatment and optimal outcome although wide excision with clean margins is highly recommended. However, more studies are needed to determine whether adjuvant treatment is necessary. Long-term surveillance is mandatory in all cases.

## References

[1] Goldenberg D, Sciubba J, Koch W, Tufano RP (2004). Malignant odontogenic tumors: a 22-year-experience. Laryngoscope.

[2] Goldenberg D, Sciubba J, Koch W, Tufano RP (2004). Odontogenic ghost cell carcinoma. Head Neck.

[3] Gorlin Rg, Pindborg JJ, Clausen FP, Vickers RA (1962). The calcifying odontogenic cyst – a possible analogue of the cutaneous calcifying epithelioma of Malherbe: an analysis of fifteen cases. Oral Surg, Oral Med and Oral Pathol.

[4] Barnes L, Eveson JW, Reichart P, Sidransky D (2005). World Health Organization classification of tumours: pathology and genetics of head and neck tumours. Lyon: IARC Press.

[5] Motosugi U, Ogawa I, Yoda T (2009). Ghost cell odontogenic carcinoma arising in calcifying odontogenic cyst. Ann Diagn Pathol.

[6] Arashiyama T, Kodama Y, Kobayashi T (2012). Ghost cell odontogenic carcinoma arising in the background of a benign calcifying cystic odontogenic tumor of the mandible. Oral Surg, Oral Med and Oral Pathol.

[7] Li BB, Gao Y (2009). Ghost cell odontogenic carcinoma transformed from a dentinogenic ghost cell tumor of maxilla after multiple recurrences. Oral Surg Oral Med and Oral Pathol.

[8] Kim Tj, Lee YS, Kim BK, Lee KY (2006). Ameloblastoma associated with dentinogenic ghost cell tumor: a case report. Korean J Pathol.

[9] Li BH, Cho Y, Kim S, Kim MJ, Hong SP, Lee JH (2011). Recurrent odontogenic ghost cell carcinoma (OGCC) at a reconstructed fibular flap: a case report with immunohistochemical findings. Med Oral Patol Oral Cir Bucal.

[10] Roh GS, Jeon BT, Parket B (2008). Ghost cellodontogenic carcinoma of the mandible: a case report demonstrating expression of tartrate-resistant acid phosphatase (TRAP) and vitronectin receptor. J Oral Maxillofac Surg.

[11] Zhu ZY, Chu ZG, Chen Y (2012). Ghost cell odontogenic carcinoma arising from calcifying cystic odontogenic tumor: a case report. Korean J Pathol.

[12] Gong YL, Wang L, Wang HK, Li T, and Chen XM (2009). The expression of NF-kB, Ki-67 and MMP-9 in CCOT, DGCT and GCOC. Oral Oncol.

[13] Folpe AL, Tsue T, Rogerson L, Weymuller E, Oda D, True L (1998). Odontogenic ghost cell carcinoma: a case report with immunohistochemical and ultrastructural characterization. J Oral Pathol Med.

[14] Piattelli A, Fioroni M, Alberti D, Rubini C (1998). Immunohistochemical analysis of a dentinogenic ghost cell tumour. Oral Oncol.

[15] Ellis GL, Shmookler BM (1986). Aggressive (malignant?) epithelial odontogenic ghost cell tumor. Oral Surg Oral Med Oral Pathol.

[16] Cheng Y, Long X, Li X, Bian B, Chen X, Yang X (2004). Clinical and radiological features of odontogenic ghost cell carcinoma: review of the literature and report of four newcase. Dentomaxillofac Rad.

[17] Lu Y, Mock D, Takata T, Jordan R (1999). Odontogenic ghost cell carcinoma: report of four new cases and review of the literature. J Oral Pathol Med.

[18] Kim HJ, Choi SK, Lee CJ, Such CH (2001). Aggressive epithelial odontogenic ghost cell tumor in the mandible: CT and MR imaging findings. AJNR Am J Neuroradiol.

[19] Grodjesk JE, Dolinsky HB, Schneider LC (1987). Odontogenic ghost cell carcinoma. Oral Surg Oral Med Oral Pathol.

[20] Ikemura K, Horie A, Tashiro H (1985). Simultaneous occurrence of calcifying odontogenic cyst and its malignant transformation. Cancer.

[21] Scott J, Wood GD (1989). Aggressive calcifying odontogenic cyst: A possible variant of ameloblastoma. Br J Oral Maxillofac Surg.

[22] McCoy BP, O'Carroll MK, Hall JM (1992). Carcinoma arising in a dentinogenic ghost cell tumor. Oral Surg Oral Med Oral Pathol.

[23] Dubiel-Bigaj M, Olszewski E, Stachura J (1993). The malignant form of calcifying odontogenic cyst: A case report. Patologia Polska.

[24] Siar CH, Ng KH (1994). Aggressive (malignant?) epithelial odontogenic ghost cell tumor of maxilla. J Laryngol Otol.

[25] Alcalde RE, Sasaki A, Misaki M (1996). Odontogenic ghost cell carcinoma: Report of a case and review of literature. J Oral Maxillofac Surg.

[26] Kamijo R, Miyaoka K, Tacbikawa T (1999). Odontogenic ghost cell carcinoma: report of a case. J Oral Maxillofac Surg.

[27] Li TJ, Yu SF (2004). Clinicopathologic spectrum of the so-called calcifying odontogenic cyst: A study of 21 intraosseous cases with reconsideration of the terminology and classification. Am J Surg Pathol.

[28] Sun ZJ, Zhao YF, Zhank L, Li ZB, Chen XM, Zhang WF (2007). Odontogenic Ghost Cell Carcinoma in the Maxilla: a case report and literature review. J Oral Maxillofac Surg.

[29] Slama A, Boujelbène N, Ben Yacoub L, Trabelsi A, Khochtali H, Sriha B (2010). Ghost cell odontogenic carcinoma of the mandible. Rev Stomatol Chir Maxillofac.

